# Proteomic Studies for the Investigation of γ-Globin Induction by Decitabine in Human Primary Erythroid Progenitor Cultures

**DOI:** 10.3390/jcm9010134

**Published:** 2020-01-03

**Authors:** Andria Theodorou, Marios Phylactides, Eleni Katsantoni, Kostas Vougas, Spyros D. Garbis, Pavlos Fanis, Maria Sitarou, Swee Lay Thein, Marina Kleanthous

**Affiliations:** 1 Molecular Genetics Thalassaemic Department, Cyprus Institute of Neurology and Genetics, Nicosia 2371, Cyprus; 2 Basic Research Center, Biomedical Research Foundation, Academy of Athens, 11527 Athens, Greece; 3 Division for Cancer Sciences, Southampton General Hospital, University of Southampton, Southampton SO16 6YD, UK; 4 Centre for Proteomics Research, Institute for Life Sciences, Highfield Campus, University of Southampton, Southampton SO17 1BJ, UK; 5 Molecular Genetics Function and Therapy Department, Cyprus Institute of Neurology and Genetics, Nicosia 2371, Cyprus; 6 Thalassaemia Centre, Larnaca General Hospital, Larnaca 6043, Cyprus; 7 Sickle cell branch, National Heart, Lung and Blood Institute, The National Institutes of Health, Bethesda, MD 20814, USA

**Keywords:** γ-globin, decitabine, primary human erythroid cultures, beta-thalassemia, HbF, iTRAQ

## Abstract

Reactivation of γ-globin is considered a promising approach for the treatment of β-thalassemia and sickle cell disease. Therapeutic induction of γ-globin expression, however, is fraught with lack of suitable therapeutic targets. The aim of this study was to investigate the effects that treatment with decitabine has on the proteome of human primary erythroid cells from healthy and thalassemic volunteers, as a means of identifying new potential pharmacological targets. Decitabine is a known γ-globin inducer, which is not, however, safe enough for clinical use. A proteomic approach utilizing isobaric tags for relative and absolute quantitation (iTRAQ) analysis, in combination with high-pH reverse phase peptide fractionation followed by liquid chromatography-tandem mass spectrometry (LC-MS/MS), was employed to investigate the effects of decitabine treatment. Bioinformatics analysis making use of the Database for Annotation, Visualization and Integrated Discovery (DAVID) was employed for functional annotation of the 192 differentially expressed proteins identified. The data are available via ProteomeXchange with identifier PXD006889. The proteins fall into various biological pathways, such as the NF-κB signaling pathway, and into many functional categories including regulation of cell proliferation, transcription factor and DNA binding, protein stabilization, chromatin modification and organization, and oxidative stress proteins.

## 1. Introduction

High levels of fetal hemoglobin (HbF) have long been recognized to be clinically beneficial in β-thalassemia and sickle cell disease, prompting numerous approaches for the reactivation of gamma globin in the treatment of these disorders. Although more than 70 HbF inducing agents have been identified so far, which can be grouped into several classes depending on their chemical structure and mechanism of action [1], their clinical use is limited due to their low HbF inducing activity, high cytotoxicity, and inability to elicit a response in all patients. Thus identification of new agents with higher efficacy and reduced toxicity is much needed.

A key step in developing new drugs is the identification of specific molecular targets whose function can be manipulated pharmacologically. Many key molecules such as BCL11a [2,3], LRF [4], and KLF1 [5,6], and pathways such as p38 MAPK [7] that mediate the induction of HbF have been identified, however, their pharmacological manipulation for the therapy of β-thalassemia has proven difficult. One potential route for identifying novel molecules for targeted drug development is to explore the molecular mechanisms of action of currently-available HbF inducers. Although the inducers themselves may not be suitable for clinical use, studying their mechanism of action may lead to the identification of key molecules which could be amenable to therapeutic manipulation. Such an approach was used in this study, which investigated the mechanism of action of HbF inducer decitabine.

Decitabine (5-aza-2’-deoxycytidine) is a cytosine analogue, which received FDA approval for the treatment of myelodysplastic syndromes in 2006. Decitabine was shown to increase HbF levels in sickle cell anemia patients non-responsive to hydroxyurea (HU) [8]. Further studies showed that decitabine could increase HbF synthesis in both baboons and sickle cell patients at much lower concentrations than its ‘parent’ compound, 5-azacytidine [8,9,10]. In addition, it increased HbF levels in β-thalassemia intermedia patients without being cytotoxic [11]. Decitabine depletes the chromatin modifying enzyme DNA methyltransferase 1 (DNMT1) by covalent trapping, leading to the hypomethylation of DNA CpG nucleotides. Although DNMT inhibitors have been known to reactivate HbF for more than 30 years, the mechanism by which they induce γ-globin expression remains controversial [1]. Studies using 5-azacytidine [12] led to the conclusion that DNMT inhibitors increase HbF production via reduction in the level of DNA methylation of the γ-globin gene promoter, however, local promoter hypomethylation is unlikely to be the sole requirement for gene expression [13]. Decitabine was also shown to specifically increase acetylation of H3 and H4 at the γ-globin promoter, suggesting that decitabine-mediated changes in DNA methylation might be associated with changes in chromatin structure [9].

To enhance our understanding of how decitabine induces HbF production, and to aid the identification of potential therapeutic targets, we employed an iTRAQ approach coupled with mass spectrometry [14] to investigate the proteome of decitabine-treated and un-treated primary human erythroid progenitor cell cultures from healthy and transfusion-dependent thalassemic donors. Comparison of the proteomic profile of treated cultures with that of un-treated cultures identified 192 significantly differentially expressed proteins and provided information on drug-mediated proteome changes and drug-induced signaling pathways that can be used as insights for therapeutic interventions.

## 2. Experimental Section

### 2.1. Primary Human Erythroid Cultures from Donors

Human erythroid progenitor cells from buffy coats obtained from healthy individuals and peripheral blood from IVS1-110/IVS1-110 transfusion-depended thalassemic donors were cultured using the two phase liquid culture described by Fibach [15]. The study was conducted in accordance with the Declaration of Helsinki and was approved by the National Bioethics Committee of Cyprus (Reference number: EEBK/EII/2013/23). Written, informed consent was provided by study participants.

### 2.2. Induction of Human Primary Erythroid Progenitor Cells

Human primary erythroid progenitor cells were seeded at ~1.5–2.0 × 10 ^6^ cells/mL and treated with 300 nM decitabine (Sigma Aldrich, St. Louis, MO, USA) on day six of Phase II. Cells grown in the absence of decitabine (un-treated samples) were used as negative controls. Cells were harvested after six days of treatment.

### 2.3. Cation Exchange High Performance Liquid Chromatography (HPLC)

The levels of HbF in primary human erythroid cultures were determined by HPLC [16] using the Schimadzu Instrument (Kyoto, Japan) with DGUA5 degasser, LC-20AD pump, SIL-20AC HT autosampler, SPD-M20A PDA detectors and CTO-20A oven. Hemoglobins were separated on a PolyCat Atm 35 mm × 4.6 mm, 5 μm, 1000 A column (PolyLC Inc., Columbia, MD, USA). The samples were eluted with a gradient of Bis-Tris-KCN-NaCl buffers and hemoglobins were detected at 417 nm [16].

### 2.4. Proteomics and Data Analysis

#### 2.4.1. Protein Digestion and iTRAQ Labeling

Cell pellets were dissolved in 200 μL dissolution buffer (0.5 M triethylammonium bicarbonate (TEAB) and 0.05% SDS). Protein concentration was determined by the Bradford assay (BioRad laboratories Inc., Hercules, CA, USA). For each sample, 100 μg of total protein underwent reduction of cysteine disulphide bonds, cysteine blockage and trypsinization as described previously [17]. Tryptic digested peptides were labelled using the 8-plex iTRAQ reagent kit (AB Sciex Inc., Foster City, CA, USA) according to manufacturer’s instructions. Following completion of the labelling reactions, samples were pooled, dried with a speedvac concentrator and stored at −20 °C until the high-pH Reverse Phase fractionation step.

#### 2.4.2. High-pH Reverse Phase (RP) Peptide Fractionation

High-pH RP C18 fractionation of the iTRAQ 8plex labelled peptides was performed on the Dionex P680 pump equipped with PDA-100 photodiode array detector using the Waters, XBridge C18 column (150 mm × 4.6 mm, 3.5 μm) as described previously [17]. The signal was monitored at 280, 254, and 215 nm and the column temperature was set to 30 °C. Fractions were collected every minute, dried through the use of a speedvac concentrator for 4–5 h and stored at −20 °C until the LC-MS analysis step.

#### 2.4.3. LC-MS Analysis

All LC-MS experiments were performed on the Dionex Ultimate 3000 UHPLC system coupled with the high resolution Thermo Orbitrap Elite mass spectrometer (Thermo Fisher Scientific, Bremen, Germany) as described previously [17]. Individual high-pH RP peptide fractions were reconstituted in 30 μL loading solution composed of 2% acetonitrile, 0.1% formic acid. Five microliters of each fraction were was injected and loaded for 8 min on the Acclaim PepMap 100, 100 μm × 2 cm C18, 5 μm, 100 Å trapping column with the ulPickUp Injection mode at a flow rate of 5 μL/min. For the peptide separation the Acclaim PepMap RSLC, 75 μm × 25 cm, nanoViper, C18, 2 μm, 100 Å column retrofitted to a PicoTip emitter (FS360-20-10-D-20-C7) was used for multi-step gradient elution. The mobile phase (A) was composed of 2% acetonitrile, 0.1% formic acid and mobile phase (B) was composed of 100% acetonitrile, 0.1% formic acid. The peptides were eluted under a 315 min gradient from 2% (B) to 33% (B). The flow rate was 300 nL/min and the column temperature was set at 35 °C. Gaseous phase transition of the separated peptides was achieved with positive ion electrospray ionization applying a voltage of 2.5 kV.

The Thermo Xcalibur software was used for spectrum acquisition and quality control. For every MS survey scan, the top 10 most abundant multiply charged precursor ions between m/z ratio 300 and 2200 and intensity threshold 500 counts were selected with FT mass resolution of 60,000 and subjected to HCD fragmentation with an isolation window of 1.2 Da. Tandem mass spectra were acquired with FT resolution of 15,000. Normalized collision energy was set to 33 and already targeted precursors were dynamically excluded for further isolation and activation for 45 sec with 5 ppm mass tolerance.

### 2.5. Database Search and Data Analysis:

The HCD tandem mass spectra collected from RP fractions were analyzed through the Proteome Discoverer software (v 1.4, Thermo, Waltham, MA, USA) for peptide and protein identifications with the following settings: MS1 Precursor Selection and spectrum properties filter (min. precursor mass 600 Da and max. precursor mass 5000 Da). All spectra were searched against a UniProt Fasta file containing 20,236 human reviewed entries. iTRAQ quantification was also performed through the same software. Protein ratios were normalized to protein median and peptides with missing iTRAQ channels were excluded from relative protein quantification. Only high confidence peptides (*p* < 0.01) were used for the analysis. Peptides were assigned to proteins using the maximum parsimony principle. The percolator module in Proteome Discoverer software (v 1.4) was used to evaluate the certainty of identifications and controlling the FDR with the following settings (Input data: Maximun Delta Cn 0.05, Decoy Database Search: Target FDR (Strict) 0.01, Target FDR (Relaxed) 0.05 and Validation based on *q*-value).

The three iTRAQ batches, maintaining the same iTRAQ labels for the same samples, were pooled and analyzed by Proteome Discoverer software (v 1.4) in a single run. Log transformation of iTRAQ ratios resulted in normal distribution as checked by Kolmogorov-Smirnof test for normality. Consequently p-values corresponding to the magnitude of differential expression were calculated from the log transformed iTRAQ ratios.

Data are available via ProteomeXchange [18] with identifier PXD006889. Supplementary Table S1 lists the proteins identified from all experiments (at Peptide level FDR ≤ 1%). Both the *q*-value and the posterior error probability (PEP), included in the Supplementary Table S2, show the confidence of the PSMs. A *q*-value is defined as the minimal false discovery rate at which the identification is considered correct. These *q*-values are estimated using the distribution of scores from the decoy database search. The PEP is the probability that the observed PSM is incorrect. Supplementary Table S2 list proteins and peptides identified from all experiments.

A *p*-value of <0.05 was used as a cut-off threshold to select the significantly differentially expressed proteins for analysis using the Database of Annotation, Visualization and Integrated Discovery (DAVID) [19].

## 3. Results

### 3.1. Decitabine Increases HbF in Primary Erythroid Cultures from Healthy Donors and Thalassemic Patients

The effect of decitabine on γ-globin gene expression was studied in primary human erythroid cultures from twelve healthy donors and eleven IVS1-110/IVS1-110 transfusion-dependent thalassemia patients. HPLC analysis showed that decitabine increased the percentage of HbF in cultures from healthy donors and thalassemic patients by an average of 62.68% and 43.89% respectively, after six days of treatment (Table 1). The increase in both sets of cultures was statistically significant (*p*-value < 0.05).

### 3.2. Proteomic Analysis of Primary Human Erythroid Cultures Revealed 192 Proteins that Are Significantly Differentially Expressed Following Treatment with Decitabine

The six (out of twelve) cultures from healthy donors and the six (out of eleven) cultures from thalassemic patients which showed the highest induction of HbF following treatment with decitabine, as determined by HPLC analysis, were selected for proteomic analysis (Table 2). iTRAQ proteomic analysis was performed for the twelve cultures, in three 8-plex experiments. Results from the three 8-plex experiments were then combined, resulting in a total of 2189 proteins being detected (Supplementary Table S1). Around 61% of the proteins (1341 out of 2189) were identified with at least two peptide matches per protein (Supplementary Table S2).

The levels of the identified proteins were compared between the treated healthy cultures and the un-treated healthy cultures (ratio 1) and the treated thalassemic cultures versus the un-treated thalassemic cultures (ratio 2). Fewer than 5% of the proteins were up- or down-regulated by more than 1.5-fold. The proteins within each comparison pair were sorted based on their log_2_ value and their *p*-values were calculated. Applying the cut-off threshold (*p*-value < 0.05), narrowed down the differentially expressed proteins to 105 proteins for the comparison between treated healthy cultures versus un-treated healthy cultures (Supplementary Table S3, Ratio 1; Table 3) and to 110 proteins between treated thalassemic cultures versus un-treated thalassemic cultures (Supplementary Table S3, Ratio 2; Table 3).

Functional annotation by DAVID of the 105 differentially expressed proteins (Table 4, Ratio 1), indicated that in healthy cultures these proteins are involved, amongst others, in transcription factor binding, regulation of cell proliferation, protein stabilization, chromatin organization, and macromolecular complex assembly. In addition, four differentially expressed proteins were associated with regulation of the NF-κB signaling pathway. Based on the functions of the up-regulated and down-regulated proteins, it can be suggested that decitabine might affect caspase-mediated apoptotic pathways and ubiquitin ligase complexes due to activation of oxidative [20] or ER stress. These findings support the positive regulation of transcription by decitabine [21].

Functional annotation analysis of the 110 differentially expressed proteins identified through comparison of treated versus un-treated thalassemic cultures showed (Table 4, Ratio 2) that the differentially expressed proteins are involved in protein and metal ion binding, DNA binding and chromatin modification and organization, transcription and positive regulation of the NF-κB cascade. Functional clustering by DAVID of the differentially expressed proteins, as well as looking into the function of individual proteins directly, suggests that decitabine is implicated in the production of immature erythrocytes. Moreover, results suggest that decitabine affects transcriptional activation of genes downstream of oxidative stress pathways through chromatin remodeling and activation of transcriptional termination and post-transcriptional processes.

Comparison of the 105 proteins in ratio 1 and the 110 proteins in ratio 2 identified 23 proteins which are common to both ratios. Therefore there is a total of 192 proteins that are significantly differentially expressed in cultures from healthy and thalassemic donors following treatment with decitabine compared to their untreated counterparts. Thalassemic cells are under considerable oxidative stress due to globin chain imbalance [20] and their expression profiles suggest that patient cells have adapted to varying degrees to these permanent stress conditions [22]. Therefore, treatment with decitabine probably modulates gene expression to a different extent in healthy and thalassemic cultures, depending on basal levels of gene expression, resulting in the relatively small overlap for ratio 1 and ratio 2 proteins.

## 4. Discussion

Traditionally, most HbF inducers have been identified based on their ability to alter local promoter chromatin (DNA methyltransferase inhibitors and Histone deacetylase inhibitors) or the kinetics of erythroid differentiation (cytotoxic agents). However, observations such as the ability of 5-azacytidine to increase HbF synthesis in the absence of global DNA methylation, the failure of γ-globin promoter hypomethylation by shRNA-mediated DNMT1 knock-down to induce expression of the gene [23], the ability of drugs to induce HbF in vitro without changes in the differentiation kinetics [23], the ability of some short chain fatty acids that do not affect histone acetylation to induce HbF synthesis [24] and the ability of p38 MAPK inhibitors to block γ-globin and HbF induction by agents [7], have raised questions about the molecular mechanism of action of some of these agents. Over the past few years, groups have redirected their research towards cell signaling as a way to regulate HbF induction. The pathways that have been demonstrated to be involved in γ-globin reactivation, so far, include cyclic guanosine monophosphate [25], cyclic adenosine monophosphate [26], nitric oxide [27], p38 MAPK [7], ROS [28], and cytokine signaling. Although there is strong evidence for the involvement of each of these signaling pathways in HbF induction, it is not clear how such a diverse group of compounds and pathways can lead to the same physiological outcome.

In the current study, we employed a quantitative proteomic approach to investigate the molecular mechanism of action of a known HbF inducer, decitabine, with the aim of identifying potential pharmacological targets that would enable the design of new drug agents. Decitabine, a DNA demethylating agent and a safer derivative of 5-azacytidine, was previously shown to increase HbF synthesis efficiently both in baboons and in patients with sickle cell disease [8,9,10]. In agreement with previous studies, we have demonstrated that decitabine can increase the percentage of HbF in primary human erythroid cultures from healthy and transfusion-dependent thalassemic donors as determined by HPLC. The increase in HbF was statistically significant after 6 days of incubation with the agent. Despite the general increase in γ-globin expression, a large variation in the response to the agent was observed between the individual cultures employed in this study. This variation in response was previously demonstrated in relation to other HbF inducers such as HU [22] and was mainly attributed to the presence of HbF-associated SNPs in the *BCL11A* gene or to the *XmnI* restriction site polymorphism of the Gγ-globin gene promoter [29,30].

The mechanism by which decitabine reactivates fetal hemoglobin in primary human erythroid cells was investigated for the first time here using the iTRAQ proteomic approach. Our results suggest that decitabine elicits HbF induction by different mechanisms in healthy and thalassemic human primary erythroid cultures.

**Figure 1:**
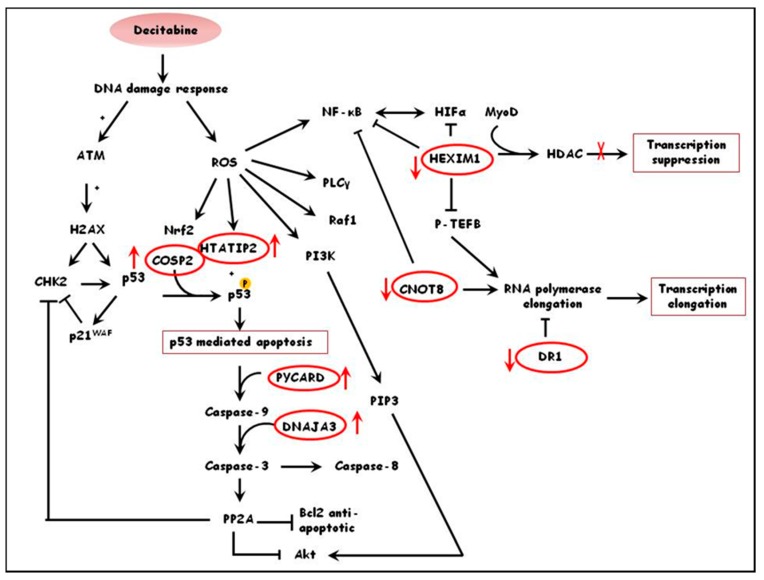
Schematic diagram demonstrating the possible effects of decitabine in primary erythroid cells from healthy donors. Decitabine promotes apoptosis through activation of stress responses, and increases transcriptional activity by downregulation of transcriptional repressors. Proteins in red circles are some of the differentially expressed proteins identified in ratio 1 (i.e., healthy treated/healthy un-treated cultures). The red arrows indicate whether the proteins are up- or down-regulated by decitabine.

Functional annotation of differentially expressed proteins in treated versus untreated healthy cultures pointed to decitabine-inducing cellular stress, such as oxidative stress, as the activator of signal transduction pathways that lead to chromatin remodeling, transcription and subsequent activation of γ-globin expression. This can be supported by the up-regulation of proteins like PYCARD, a mediator of caspase 8 and 9-mediated apoptosis [31], COPS2, a regulator of cullin-based ubiquitin E3 ligase complexes that mediates p53 degradation [32], DNAJA3, modulator of apoptotic signal transduction [33], and HTATIP2, a tumor suppressor involved in cellular oxidative stress surveillance which induces p53 mediated apoptosis [34,35]. Furthermore, differentially expressed proteins show that decitabine increases transcription in healthy cultures as seen by the down regulation of transcriptional inhibitors including DR1, a TATA-binding protein-associated phosphoprotein inhibitor of basal and activated gene transcription [36], CNOT8, a subunit of CCR4-NOT complex which regulates RNA polymerase II transcription and cellular proliferation [37,38] and HEXIM1, an inhibitor of positive transcription elongation factor and subsequently transcription elongation of RNA polymerase II [39].At the same time decitabine up-regulates proteins that promote transcription such as MYBL1, a transactivator of promoters containing Myb-binding sites [40,41]. A hypothetical pathway integrating some of the proteins mentioned and indicating the possible effect on decitabine on healthy erythroid precursor cultures is shown in Figure 1.

The above analysis agrees with claims that increasing levels of stress, such as in cases of hemolysis and hypoxia, lead to increased HbF contents per F cell due to stimulation of maturation of erythroid progenitors that retain the ability to produce HbF [42,43]. Multiple studies have shown that decitabine increases γ-globin expression by post-transcriptional and/or translational mechanisms associated with activation of stress-signal transduction pathways rather than through direct transcriptional activation [1,23,44]. Moreover, decitabine was recently shown to induce ROS generation in leukemia cells due to hypomethylation-independent incorporation of decitabine into DNA with subsequent activation of signaling pathways due to DNA damage [45]. Potentially, this type of DNA damage by decitabine leads to the cellular stresses observed in the healthy primary erythroid cultures. Moreover, the toxic effects of 5-azacytidine, the compound from which decitabine is derived, were suggested to be responsible for the augmentation of HbF synthesis due to accelerated maturation of early progenitors [46].

**Figure 2:**
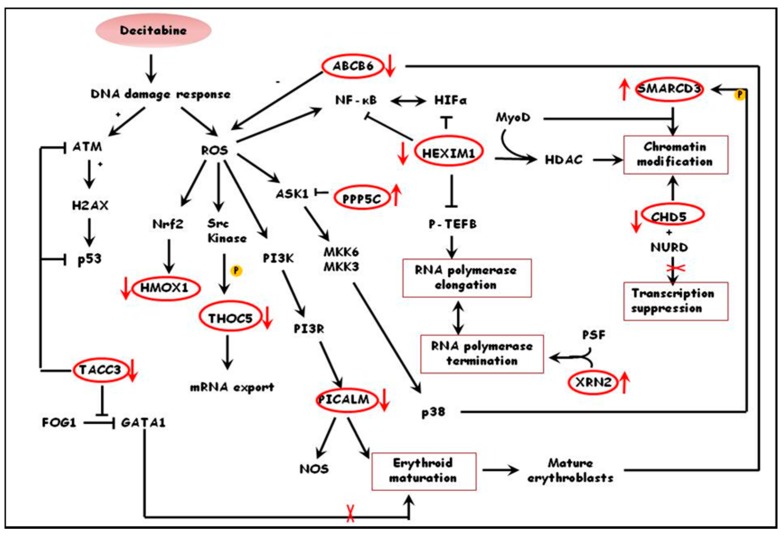
Schematic diagram demonstrating the possible effects of decitabine on primary human erythroid cells form thalassemic donors. Decitabine promotes transcription through down-regulation of transcriptional repressors and through chromatin modifications, rather than activation of oxidative stress pathways. In addition, decitabione favors immature erythroblasts and protects them against stresses. Proteins in red circles are some of the differentially expressed proteins in thalassemic cultures treated with decitabine (ratio 2). The red arrows indicate whether the proteins are up- or down- regulated by decitabine.

Similar functional annotation of differentially expressed proteins in treated and un-treated thalassemic cultures indicated that in these cultures decitabine promoted survival of immature erythroid progenitors, possibly due to modulation of erythropoiesis. This is supported by the down-regulation of proteins involved in erythroid maturation such as PICALM, a clathrin assembly lymphoid myeloid leukemia protein involved in erythroid maturation and transferrin internalization in mice [41], ABCB6, a glycoprotein expressed in the membrane of mature erythrocytes and in exosomes released from reticulocytes at the final steps of erythroid maturation [47] and up-regulation of TACC3, a protein highly expressed in hematopoietic progenitors [48]. Moreover, results suggest that decitabine affects transcriptional activation of genes downstream of oxidative stress pathways such as HMOX1, a target gene of the central regulator of cellular oxidative stress response Nrf2 [49] and up-regulation of THOC5, a key protein in the maintenance of hematopoietic stem cells which is phosphorylated in the presence of oxidative stress [50] and PPP5C, a negative regulator of hypoxia-induced activation of apoptosis signal-regulating kinase 1 [51] Proteins such as RBM10, an RNA binding regulator of splicing [52] and XRN2, a 5’-3’ exonuclease that promotes transcriptional termination [53,54] were upregulated, while HEXIM, a regulator of transcriptional elongation of RNA polymerase II [39] was downregulated. These findings suggest that decitabine promotes transcriptional termination and post-transcriptional processes. Decitabine also appears to regulate chromatin remodeling as can be observed by the up-regulation of SMARCD3, a subunit of SWI/SNF complex that promotes DNA-histone dissociation [55] and the downregulation of CHD5, a chromatin remodeling, helicase and DNA-binding protein which is activated by demethylation of its promoter [56]. A hypothetical pathway of the effects of decitabine treatment of thalassemic cultures is shown in Figure 2.

Interestingly, functional annotation of the differentially expressed proteins indicated the involvement of decitabine with the NF-κB pathway in both healthy and thalassemic cultures. Among the differentially expressed proteins, ARHGAP4, EGLN2, and USP11 were some of the factors involved in the NF-κB pathway. Treatment of healthy erythroid cultures with decitabine led to a down-regulation of ARHGAP4 and EGLN2 and upregulation of USP11 Several lines of evidence have suggested that NF-κB plays a crucial role in erythropoiesis. NF-κB is active during the early stages of normal erythroid development [57,58] and is responsible for the suppression of a number of transcription factors critical for normal erythropoiesis including MYB, MYC, and NFE2. Liu et al. [59] demonstrated that activation of the NF-κB pathway represses the expression of the α-like globin genes through repression of the erythroid-specific subunit p45 of NF-E2, a DNA-binding transcription activator that binds to the HS-40 enhancer and the LCR of the β-globin locus. Resveratrol was shown to inhibit TNFα-mediated NF-κB activation and promote erythropoiesis in primary erythroid cells [58], while thalidomide induces HbF production possibly through suppression of NF-κB induction by inflammatory cytokines such as tumor necrosis factor, which is associated with increased release of ROS. In the case of decitabine, it is possible that the NF-κB pathway may modulate γ-globin gene expression through regulation of erythropoiesis by targeting c-myb, c-myc, and NF-E2, and/or NF-κB modulation may be a secondary response to cellular stress or upstream signal transduction pathways.

This paper used iTRAQ methodology to investigate the changes in the proteome of human primary erythroid progenitor cell cultures from healthy and β-thalassemic donors, following treatment with the HbF inducer decitabine. Investigation of the differentially expressed proteins identified through this investigation may lead to the discovery of novel therapeutic targets for the treatment of β-thalasemia and related hemoglobinopathies. Functional annotation analysis of the resulting proteins points to cellular stress playing a role in HbF activation by decitabine in healthy cultures, whilst modulation of erythropoiesis features in the induction of thalassemic cultures. NF-κB may be implicated in HbF induction in both types of cultures. Potential pathways incorporating some of the proteins identified have been presented but these need to be validated through functional studies.

## Figures and Tables

**Table 1:** The effect of decitabine induction on human erythroid progenitor cell cultures. The effect of decitabine on the levels of fetal hemoglobin levels in primary human erythroid progenitor cells from healthy and thalassemic donors. Fetal hemoglobin (HbF) levels were determined after 6 days of treatment with decitabine (Dec). The results are the average of 12 healthy cultures and 11 thalassemic cultures.

Samples	HbF Levels (% Relative to Un-Treated Control)	*p*-Value (Paired *t*-Test ± Dec)
Healthy	62.68 ± 28.66	0.00003
Thalassemic	43.89 ± 38.72	0.0027

**Table 2:** HbF induction data for the primary erythroid cultures from healthy and thalassemic donors selected for proteomic analysis. For each culture, the HbF levels before treatment with decitabine (un-treated) and after six days of treatment with decitabine are shown. (**A**) Data for cultures from healthy controls and (**B**) form thalassemic patients.

**(A)**			
**Healthy Samples**			
**Culture ID**	**HbF (%)**		**Increase in HbF (% Relative to Un-Treated)**
	**Un-treated**	**300 nM Dec**	
H1	3.74	8.01	114.2
H2	10.3	19.65	90.89
H3	5.65	8.86	56.83
H4	12.83	19.73	53.78
H5	5.07	9.1	79.51
H6	2.53	4.22	66.78
Average	6.69	11.6	77
**(B)**			
**Thalassemic Samples**			
**Culture ID**	**HbF (%)**		**Increase in HbF (% Relative to Un-Treated)**
	**Un-Treated**	**300 nM Dec**	
Th1	21.79	31.35	43.88
Th2	15.8	22.65	43.35
Th3	18.11	45.21	149.68
Th4	23.48	36.67	56.13
Th5	9.87	15.46	56.64
Th6	17.61	27	53.29
Average	17.78	29.72	67.16

**Table 3:** Numbers of significantly differentially expressed proteins. The number of statistically significant differentially expressed proteins identified through comparison of the different culture conditions (treated healthy cultures versus un-treated healthy cultures (ratio 1), treated thalassemic cultures versus un-treated thalassemic cultures (ratio 2)). Expression ratios represent the fold change in protein expression.

	Number of Proteins with *p*-Value < 0.05	Up-Regulated Proteins				Down-Regulated Proteins		
		Number of Proteins	% Proteins with Expression Ratio < 1.2	1.2 < % Proteins with Expression Ratio > 2	% Proteins with Expression Ratio > 2	Number of Proteins	% Proteins with Expression Ratio < 0.8	% Proteins with Expression Ratio < 0.5
**Ratio1**	105	47	0	95.7	4.3	58	100	0
**Ratio2**	110	55	65.5	25.5	9.1	55	92.7	7.2

**Table 4:** Functional analysis of the differentially expressed proteins using Database for Annotation, Visualization and Integrated Discovery (DAVID). Bioinformatic analysis of the 105 significantly differentially expressed proteins in response to decitabine in human primary erythroid cultures from healthy donors (Ratio 1) and the 110 significantly differentially expressed proteins in response to decitabine in human primary erythroid cultures from thalassemic donors (Ratio 2). Functional analysis by DAVID of significantly differentially expressed proteins (ratios with *p*-value < 0.05) using as a reference list of all 2189 proteins identified, grouped proteins according to their biological process (BP), molecular function (MF), and cellular component (CC). In DAVID, protein groups are categorized according to their enrichment scores and corresponding *p*-value and Benjamini values.

	Functional Clustering	Enrichment Score	Count	*p*-Value	Benjamini
**Ratio 1**					
Transcription factor binding	MF	1.56	11	0.0045	0.75
Regulation of cell growth	BP	1.4	5	0.0081	0.94
Regulation of cellular component size	BP	1.4	7	0.015	0.89
Regulation of protein localization	BP	1.32	5	0.012	0.88
Lipid transport	BP	1.32	6	0.0027	0.94
Focal adhesion	CC	1.13	5	0.02	0.74
Kinase inhibitor activity	MF	0.89	3	0.068	0.91
Protein stabilization	BP	0.92	4	0.0092	0.91
Protein kinase cascade	BP	0.79	6	0.035	0.98
Chromatin organization	BP	0.74	8	0.047	0.96
Transcription cofactor activation	MF	0.72	8	0.012	0.83
Macromolecular complex assembly	BP	0.7	15	0.038	0.97
Regulation of I-kappaB kinase/NF-kappaB cascade	BP	0.66	4	0.055	0.97
Regulation of transferase activity	BP	0.61	6	0.039	0.96
**Ratio 2**					
Transition metal ion binding	MF	1.52	19	1.70 × 10^2^	9.90 × 10^1^
Zing finger	MF	1.52	10	2.00 × 10^2^	9.80 × 10^1^
Cerebral cortex development	BP	1.35	3	2.50 × 10^2^	9.80 × 10^1^
Chromatin organization	BP	1.35	12	2.00 × 10^4^	1.70 × 10^1^
Transcription	BP	1.29	14	2.10 × 10^2^	9.90 × 10^1^
Gamete generation	BP	1.13	6	4.10 × 10^2^	9.50 × 10^1^
Chromatin modification	BP	0.98	6	2.30 × 10^2^	9.90 × 10^1^
Positive regulation of I-kappaB kinase/NF-kappaB cascade	BP	0.95	4	3.60 × 10^2^	9.50 × 10^1^

## Supplementary Materials

The following are available online at https://www.mdpi.com/2077-0383/9/1/134/s1, Table S1: Table of all the proteins identified from all experiments (at Peptide level FDR ≤ 1%). In total, 2188 proteins were identified from all three experiments. The table lists the percentage of coverage of each protein identified along with the number of proteins, the number of unique peptides, and the number of peptide spectrum matches (PSM) used for identification of each protein. The number of PSMs is the total number of identified peptide spectra matched for each protein. Proteins were expressed in ratios of treated (300 nM Decitabine) versus un-treated. Samples from healthy primary cultures were labelled using 113, 115 tags (un-treated), and 114, 116 (treated), whereas for samples from thalassemic primary cultures, tags 117, 119 (un-treated), and 118, 121 (treated) were used. Therefore, for healthy cultures, each protein was expressed in ratios of (114 + 116)/(113 + 115) and for thalassemic cultures in ratios of (118 + 121)/(117 + 119). Each protein is given a score which is the sum of the scores of the individual peptides. The score represents a search-dependent method of scoring the number of fragment ions that are common to two different peptides with the same precursor mass and calculates the cross-correlation score for all candidate peptide, Table S2: Table of the proteins and their corresponding peptides identified from all three iTRAQ experiments. The table lists the coverage percentage along with the number of proteins and unique peptides used for identification of each peptide. As in Supplementary Table S1, the peptides and consequently proteins are expressed in ratios of treated (300 nM decitabine) versus un-treated in both healthy ((114 + 116)/(113 + 115)) and thalassemic ((118 + 121)/(117 + 119)) cultures. Both the q-value and the posterior error probability (PEP), included in the table, show the confidence of the peptide spectrum match (PSMs). A *q*-value is defined as the minimal false discovery rate at which the identification is considered correct. These *q*-values are estimated using the distribution of scores from the decoy database search. The PEP is the probability that the observed PSM is incorrect, Table S3: Table of proteins that are significantly differentially expressed for each comparison of culture conditions (ratios 1 and 2). The proteins are listed following sorting based on their Log_2_ values and selection of proteins with a *p*-value < 0.05. (Ratio 1: Differentially expressed proteins in treated compared to un-treated healthy samples. Ratio 2: Differentially expressed proteins in treated compared to un-treated thalasemic cultures. Protein ID refers to the Uniprot Assession number corresponding to each protein. A * is used to mark proteins which are common between Ratio 1 and Ratio 2. Protein description lists the full name of the protein. Protein name refers to the acronym of the protein which usually corresponds to the gene name. Average ratio corresponds to the average of the differential expression of the protein from three experiments. Log_2_ value is the log_2_ value of the average ratio and the *p*-value states the statistical significance of the log_2_ value of each protein. The coverage is the percentage of the protein sequence that was identified through peptides and MW corresponds to the molecular weight of each protein.
